# Dr. Fordyce Barker on the Value of Permanganate of Potash in the Treatment of Amenorrhœa

**Published:** 1886-05

**Authors:** 


### Dr. Fordyce Barker on the Value of Permanganate of Potash in the Treatment of Amenorrhœa.

It has been some five years since Drs. Ringer and Merrell first called the attention of the profession to the value of manganese in the treatment of amenorrhœa and other disturbances of the uterine functions. Since this announcement was made, this drug has been employed by a number of careful observers in the treatment of the conditions mentioned, and the reports which have been published have tended to confirm the favorable opinion expressed by Drs. Ringer and Merrell. The drug has undoubtedly disappointed some practitioners who have used it, and adverse reports have been a natural result of its indiscriminate employment. There are certain conditions of the generative function in which amenorrhœa is but a natural result, conditions in which manganese is as likely to fail in its duty as any other of the numerous so-called emmenagogues which are employed. Every one who has had a large experience with those articles of the materia medica which are classed as emmenagogues must have realized the fact that in certain cases these agents will fail, and the uterine function will remain uninfluenced by their action. It is, perhaps, in this class of cases that manganese will be found of little value in establishing the periodic flow of blood from the uterus. There can be but little doubt, however, of the fact that manganese is almost specific in its action in overcoming amenorrhœa in a large number of cases. Proof of this assertion will appear when the literature of this subject is carefully studied. We have before us a very valuable contribution to this subject from the pen of Dr. Fordyce Barker, of New York (*New York Medical Journal*, February 27, 1886), in which an experience is related which greatly strengthens this statement. Dr. Barker has used the permanganate of potash almost exclusively, when an emmenagogue was indicated, for the past four years. The class of cases in which he has employed it are arranged in the following groups :

" First.—Young ladies between the ages of fourteen and nineteen, who come from the country 'to finish their education.' Home-sickness, entire change of their habits of life and associations, overtax of their brain power from their own or their teachers' ambition to accomplish more in a given time than they ought to attempt, not infrequently lead to an arrest of menstruation. I see at least ten or fifteen such patients every winter.

" Second.—Ladies, both young and married, who suffer severely from seasickness, that have left some European port within a few days of the menstrual period. With such, amenorrhœa, of longer or shorter duration, is almost sure to follow. I am consulted by at least eight or ten such every year.

" Third.—Ladies between thirty and forty, generally married, some of whom have borne children, who rapidly begin to gain flesh, grow stout, while, at the same time, menstruation decreases in both duration and quantity, until at last it is only a mere pretense. This is generally attended with annoying nerve disturbances, pelvic weight, sometimes hæmorrhoids, and often mental depression, from the apprehension of growing old prematurely."

Dr. Barker now avows that never, where in either of these classes of cases that he has prescribed the premanganate, has he *known* it to fail. In prescribing the permanganate, he directs the patients to continue the use of the medicine, if necessary, for at least three months, and he especially urges them to report to him, either personally or by letter, if the end is not accomplished. With this specific treatment, Dr. Barker has, however, not neglected any other measures necessary to keep up a healthy and regular action of other functions. He is also careful never to prescribe the permanganate in cases where the amenorrhœa is due to some grave constitutional disease, nor does he rely on it for the relief of sudden suppression due to cold, moral shock, or an acute disease.

Dr. Barker has experienced the same difficulties in administering the permanganate as have been reported by others who have prescribed the drug. It is well known that the permanganate undergoes rapid decomposition, and that it will produce, in most persons, severe gastric pains. Indeed, it is taken with great repugnance, as a rule. To overcome the peculiar taste and unpleasant effects of the drug, Dr. Barker has adopted the plan of giving it in two-grain tablets, and immediately thereafter administering a half-tumbler of water, not cold. He usually prescribes two grains three times a day. Dr. Barker gives, as the result of his clinical experience with the permanganate of potash, a report of forty-three cases of amenorrhœa, of the groups mentioned, and, at the time of writing, he is not able to recall an instance where he made a domicile visit for this disease.

It is well known that Dr. Barker is an exceedingly careful observer and accurate reporter. His experience with the permanganate in amenorrhœa speaks volumes in favor of its judicious employment in the groups of cases in which he has obtained his results.—*Editorial in Maryland Medical Journal.*

